# Editorial: Non-intubated thoracic surgery: From concepts to clinical reality

**DOI:** 10.3389/fsurg.2022.948373

**Published:** 2022-08-02

**Authors:** József Furák, Davide Tosi

**Affiliations:** ^1^ Department of Surgery, Faculty of Medicine, University of Szeged, Szeged, Hungary; ^2^ Thoracic Surgery and Lung Transplantation Unit, Fondazione IRCCS Ca’ Granda, Ospedale Maggiore Policlinico, Milan, Italy

**Keywords:** thoracic surgery, anesthaesia, non-intubated thoracoscopic lung surgery, video-assisted thoracic surgery (VATS), lung cancer

Editorial on the research topic Non-intubated thoracic surgery: From concepts to clinical reality by Furák J, and Tosi D. (2022). Front. Surg. 0:000. doi: 10.3389/fsurg.2022.948373

As non-intubated thoracic surgery (NITS) has become a popular procedure in dedicated centers and has proved to be better than others, clinicians have realized that despite its advantages, the widespread utility of this procedure has not yet become a reality. Many thoracic surgeons and anesthesiologists have resisted applying this procedure citing some reasons such as an unsafe airway, the difficult conversion to the intubation procedure if necessary, and the possible problems related to gas exchange. However, also given the fact that many thoracic surgeons are highly satisfied with NITS, herein, we thought it appropriate to discuss the most frequently emerging questions regarding its difficulties. We hope that those who are not convinced about the importance of this procedure can gain some information to resolve their doubts and misgivings. We attempt to deal with the dark side of NITS and clear the pink fog around NITS by shedding light on its real practice.

We would like to introduce readers to some of the most relevant articles.

The first one is a basic article: *Pro and Cons of the NITS*, which presents the current clinical practice of NITS. The authors emphasize the advantages and disadvantages of NITS vis-à-vis the other thoracic surgeries performed under general anesthesia. They show that the surgical technique of NITS is very similar to that of the intubated one, but that anesthesia plays a massive role during NITS. They conclude that in most cases, the standard procedures in thoracic surgery are done under general anesthesia, but in selected cases in selected centers, the non-intubated procedure can be performed as an alternative (Janik et al. 2021).

The next article *Conversion from NITS to intubated procedure* discusses one of the most important and debated points of NITS and provides the current state of the art. Here, the reader can gain information on incidence, indications, techniques, and prevention of conversion. Because the authors have significant experience with performing NITS, the presented information is very practical. They emphasize that the team, including surgeons and anesthesiologists, must be experienced with NITS to be able to perform a successful conversion. One of the most important sections of this article is the discussion on how to prevent the need for conversion with proper selection. The authors emphasize that if conversion is necessary in an emergency situation, it must be performed in an earlier phase of the surgery and not in a later one (Chiang et al. 2021).

*The anesthesiologist's perspective regarding non-intubated thoracic surgery: a scoping review* reports the results of an analysis of 53 primary studies about NITS, published between 2011 and 2021, giving a complete overview of the current state of NITS, particularly of the anesthesiological aspects (Rosboch et al. 2022).

Because anesthesia is a key point of spontaneous ventilation thoracic surgery, we would like to recommend an article on anesthesia for surgeons. As discussed before, NITS is a team effort, which is why a surgeon must have information about the nature of work on the other side of the lira. *Anesthesiological Aspect* summarizes the current theoretical and practical knowledge about spontaneous-ventilation thoracic surgery. The authors mention an interesting type of spontaneous ventilation, in which a double lumen tube is inserted into the trachea, giving a safe airway (Fabo et al. 2022).

The negative effects of the mechanical one-lung ventilation procedure are extensively discussed in *Pathophysiological advantages of spontaneous ventilation*. The authors focus on the mid- and long-term benefits of less postoperative dysregulation in inflammation and immunity after NITS, compared with standard operations (Lantos et al. 2022).

Probably, NITS can be viewed as a double option for patients: for those who cannot tolerate general anesthesia in minor surgery and for those fit for major surgery. The aspects of patient selection in relation to the choice of surgical procedure are comprehensively discussed in two articles by highly expert teams: *Nonintubated thoracic surgery: wedge resections for peripheral pulmonary nodules* (Ambrogi et al. 2022) and *Lung biopsy with a non-intubated VATS approach in an obese population: indications and results* (Cherchi et al. 2022).

Unfortunately, to date, it was infrequent to find dedicated NITS teams due to a lack of specific information on the procedure and the persistence of skepticism toward this method. It requires an investment of both time and energy in creating a dedicated functioning team, which has to be maintained over time.

We are confident that after the reader has read these articles, they will be convinced of the safety of NITS, which causes less surgical trauma in patients undergoing thoracic surgery. During NITS, it is imperative that both surgeons and anesthesiologists work together as their work is allied with each other. With this teamwork, patients can benefit from the real advantages of spontaneous ventilation thoracic surgery.

